# From Waste to Wealth: Exploring the Bioactive Potential of Wine By-Products—A Review

**DOI:** 10.3390/antiox13080992

**Published:** 2024-08-15

**Authors:** Glenda-Caridad Peña-Portillo, Sergio-Miguel Acuña-Nelson, José-Miguel Bastías-Montes

**Affiliations:** Departamento de Ingeniería en Alimentos, Universidad del Bío-Bío, Avenida Andrés Bello 720, Chillán 3780000, Chile; glenda.pena2201@alumnos.ubiobio.cl (G.-C.P.-P.); jobastias@ubiobio.cl (J.-M.B.-M.)

**Keywords:** biowaste, wine industry, polyphenolic compounds, extraction methods

## Abstract

The present paper explores the biological potential of bioactive compounds present in wine industry wastes, highlighting their valorization to promote sustainability and circular economy. Wine by-products, such as grape pomace and vine shoots, contain a high concentration of polyphenols, flavonoids, anthocyanins and other phytochemicals with antioxidant, anti-inflammatory and anticarcinogenic properties. Both conventional extraction methods, such as solid–liquid extraction, and emerging technologies, including enzyme-assisted extraction, ultrasound-assisted extraction, supercritical fluid extraction, microwave-assisted extraction, pressurized liquid extraction, high-hydrostatic-pressure extraction, and deep natural solvent-assisted extraction (NaDES), are discussed. In addition, the preservation of polyphenolic extracts by microencapsulation, a key technique to improve the stability and bioavailability of bioactive compounds, is addressed. The combination of advanced extraction methods and innovative preservation techniques offers a promising perspective for the valorization of bioactive compounds from wine residues, driving sustainability and innovation in the industry.

## 1. Introduction

At the intersection of food science research and biochemistry, a fascinating and increasingly relevant field of study is emerging: the bioaccessibility and bioavailability of bioactive compounds present in biowaste from the wine industry. This field of research is immersed in the complexity of understanding how bioactive compounds essential for human health can be effectively extracted from these wastes, and once released, how they become accessible and usable by the organism [1].

Bioaccessibility, considered the fraction of compounds that is released and available for absorption, together with bioavailability, which describes the amount of these compounds that eventually reach the bloodstream and is available for physiological utilization, are key concepts in this scientific exploration. Addressing these concepts involves not only defining them rigorously but also understanding the underlying mechanisms that determine their magnitude and relevance [2].

In this review, we focus on the specificity of bioactive compounds from wine industry biowaste, exploring the molecular diversity that characterizes these by-products of winemaking. In addition, methods of extraction and release of these compounds are examined, evaluating their efficacy and contribution to the overall bioaccessibility process.

The crucial question about the importance of bioaccessibility and bioavailability in the context of human health leads to reflection on the importance of these processes to optimize the use of bioactive compounds present in biowaste from the wine industry. Scientific analysis of these processes is expected to contribute to the understanding and development of strategies that maximize the beneficial potential of these resources, opening new perspectives at the intersection between winemaking and human health.

## 2. An Analysis of the Biological Potential of Bioactive Compounds

### 2.1. General Information

Plant foods, including fruits, vegetables and processed foods, contain numerous types of phytonutrients such as vitamins, carotenoids, polyphenols, curcuminoids, polyunsaturated fatty acids, proteins, peptides, dietary fibers, oligosaccharides and minerals, which provide many beneficial effects to human health. However, the increase in synthetic foods, as well as the use of food additives, has generated a growing interest in natural products for a balanced and healthy diet. Therefore, a great deal of research has been focused on the search for functional natural products with high nutritional value and positive health effects [3,4,5,6,7,8,9,10,11].

Bioaccessibility and bioavailability are fundamental concepts in the field of nutrition and toxicology. These terms refer to the amount and rate at which a compound or nutrient is absorbed and utilized by the human body [12]. Bioaccessibility corresponds to the fraction of a compound or nutrient that is released and available for absorption in the gastrointestinal tract, that is, how nutrients pass through the digestion process and how they are released from food so that they can be absorbed by our body [12]. Bioaccessibility can be affected by factors such as the way the food is cooked and roasted, the food matrix, and the presence of certain chemical compounds [3,13,14,15].

Bioavailability is the amount of a compound or nutrient that is absorbed by the body and is available for utilization and metabolization in the tissues [12]. This includes how nutrients are absorbed through the intestinal cells and then transported through the blood to different parts of the body. Bioavailability may depend on factors such as the efficiency of intestinal absorption and the presence of other nutrients or compounds that may interact and compete for absorption, but there is no dependence between the amount of these compounds and their bioavailability in the human body [13,16].

There are important factors related to the intestinal microbiome that affect the bioavailability, digestion, and absorption of food. Hence, it is crucial to determine the changes due to exposure to bioactive compounds in the gastrointestinal tract [3,17]. Most of the bioactive food compounds with a positive impact on health come from plants, with animal sources being a minority in this respect [13].

As mentioned, bioactive compounds have emerged as fundamental agents in the promotion of human health. There are diverse sources of compounds, ranging from plants to microorganisms. Plants have historically been rich sources of bioactive compounds in which the synthesis and accumulation of these compounds are influenced by genetic, environmental and management factors. Manipulation of agronomic and technological factors can modulate the concentration and bioavailability of these compounds in foods [18]. Microorganisms, both bacteria and fungi, have proven to be rich sources of bioactive compounds with medicinal applications [19,20]. For example, *Streptomyces* strains have produced antibiotics such as streptomycin [21]. In addition, fungi of the genus *Penicillium* have given rise to penicillin, revolutionizing antimicrobial medicine [20].

Among these bioactive food compounds with a positive impact on health are carotenoids, which are found in red, orange, yellow, and dark green fruits and vegetables, such as carrots, tomatoes, and spinach. They have antioxidant properties and have been shown to help reduce the risk of eye diseases such as macular degeneration and cataracts, as well as cardiovascular diseases. Likewise, fatty acids (omega 3) can be found in foods such as flaxseeds, chia seeds and walnuts. Fatty acids have anti-inflammatory properties and have been shown to help reduce the risk of cardiovascular disease, improve brain health, and reduce inflammation in the body [22,23,24,25].

Soy and its by-products are rich in isoflavones. These have estrogen-like effects and have been shown to help relieve menopausal symptoms, reduce the risk of cardiovascular disease and osteoporosis, and have anticancer properties. Phytosterols and stanols present in plant foods such as nuts, seeds, vegetable oils, and legumes are compounds that help reduce blood cholesterol levels, which may contribute to the prevention of cardiovascular disease [26,27,28].

Polyphenolic compounds can be found in fruits, vegetables, tea, red wine, among other foods, as well as in many biowastes. They have antioxidant, anti-inflammatory and neuroprotective properties, and have been associated with reducing the risk of cardiovascular disease, cancer and neurodegenerative diseases. They have also been shown to help lower blood pressure and reduce the risk of chronic diseases such as type 2 diabetes [29,30,31,32,33]. Similarly, phenolic substances exhibit antibacterial properties, presumably due to iron deprivation or hydrogen bonding with microbial enzymes or vital proteins [34].

In the particular case of polyphenols, their bioavailability is influenced by multiple factors, ranging from digestion and absorption processes to interaction with other nutrients, individual variability and physiological or pathological conditions.

Various industrial by-products and biowaste are good sustainable sources of phytochemicals that can be valorized into bioactive compounds [35]. Since food waste is generally rich in organic components and moisture, it should not be treated by conventional methods such as incineration and landfilling. Therefore, untreated waste becomes a breeding ground for a wide variety of pathogens [36,37].

The circular economy is therefore based on the principles of reducing, reusing, recycling, and recovering the materials used, as well as reusing waste by interconnecting water, food, and energy resources [36]. This is based on three basic principles applicable to the circular economy: preserving and enhancing natural capital; optimizing resource efficiency; promoting the efficiency of systems; and promoting the use of natural resources [38].

Although grape pomace is considered a residue or waste, it is usually used either as animal feed or as fertilizer for the grape plantations themselves. Providing added value to this by-product is more than four decades old. Its valorization is based on its use as a potential source for obtaining bioactive compounds, and it is also considered a strategy for reducing environmental pollution [39].

Understanding and taking these factors into account is essential to maximize the efficiency of polyphenolic compound extraction and to take advantage of their potential health benefits.

### 2.2. Bioactive Compounds Present in Biowaste from the Wine Industry

Studies affirm that grape juice is a very good source of polyphenolic compounds, and the absence of alcohol makes it a product of mass consumption, especially for children, the elderly, and pregnant women [40]. However, these compounds can also be found in residues generated in processes in which grapes are the raw material.

**Figure 1:**
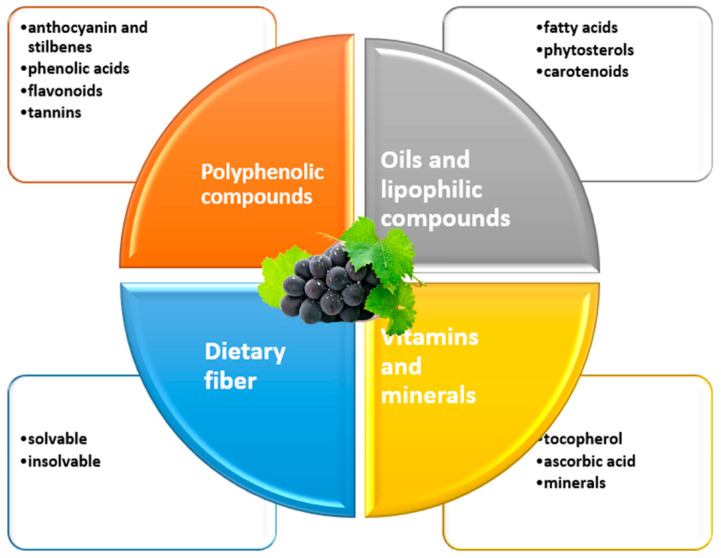
Bioactive compounds in grape pomace (skin, seed, pulp and stalk).

The wine industry is one of the main agri-food activities worldwide, generating a significant number of by-products and waste during its production process. These biowastes, which mainly include skins, seeds, and grape residues, are used in the production of wine [41,42] and represent a valuable source of bioactive compounds, with potential benefits for human health and applications in various areas, from food to medicine and cosmetics. According to the literature reviewed, the benefits of these bioactive compounds can be classified into four major areas, as shown in Figure 1.

In particular, polyphenols give foods and beverages derived from plants their main sensory characteristics, highlighting color and properties related to flavor, bitterness or astringency [43,44]. In addition, their versatile chemical structure confers antioxidant and anti-inflammatory properties, which have led to growing interest in their beneficial role in human health. Scientific research has shown that polyphenols can contribute to the prevention of chronic diseases, such as heart disease, cancer and diabetes, while supporting immune system and brain health [1]. Polyphenols constitute a large group of bioactive phytochemicals, of which more than 8000 molecules have been described, including multiple subclasses such as flavonoids, stilbenes, phenolic acids, and lignans [45,46].

Among the valuable bioactive compounds extracted from plant material, flavonoids are known as polyphenolic secondary plant metabolites and therefore antioxidants. They exhibit various effects such as anti-aging, anti-inflammatory, cardioprotective, immunomodulatory, antiviral, antibacterial and antifungal properties [47].

**Figure 2:**
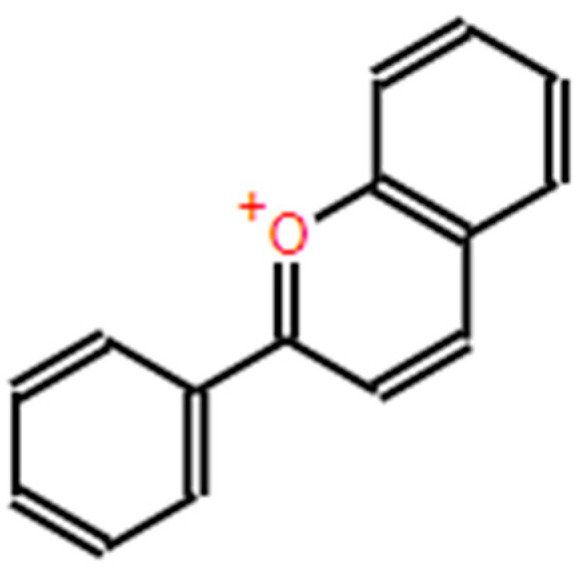
Anthocyanin’s structure.

Anthocyanins belong to the flavonoid group. They are responsible for the red, purple or blue color of grapes and other fruits. These compounds have a basic C-skeleton of C6–C3–C6 as shown in Figure 2, according to software [48] https://Biomodel.Uah.Es/En/DIY/JSME/ (accessed on 28 June 2024), and are constituted by an anthocyanidin molecule, which is the aglycone, to which a sugar is attached through a β-glycosidic bond. The basic structure contains a flavone core consisting of two aromatic rings: a benzopyrilium (A) and a phenolic group (B), both linked by a three-carbon unit [49,50].

The most common anthocyanins are cyanidin, delphinidin, pelargonidin, peonidin, petunidin and malvidin. Studies demonstrate their significant antioxidant properties, and they have been shown to have beneficial anti-inflammatory, anti-tumor (breast, liver and colon), anti-diabetic and cardiovascular effects. In addition, it has been suggested that anthocyanins may have positive effects on eye health, as well as strong antimicrobial activity [51,52].

When talking about wine, one of the sensory characteristics that stands out, especially in red wines, is their astringency; this property is mainly due to the tannins present in the grapes. Tannins are high-molecular-weight polyphenols and are found in large quantities in the skins, seeds, and stems of grapes [53,54,55,56]. Tannins include substances that are not only grouped by structural analogy but also by common characteristics. These are divided into two groups: hydrolysable tannins and condensed tannins [57,58].

Hydrolysable tannins are esters of different phenolic acids (gallic and ellagic acid). In general, they constitute complex mixtures with different phenolic acids esterified in different positions. They are characterized by being very soluble in water, especially in hot water, forming colloidal solutions [57,58].

**Figure 3:**
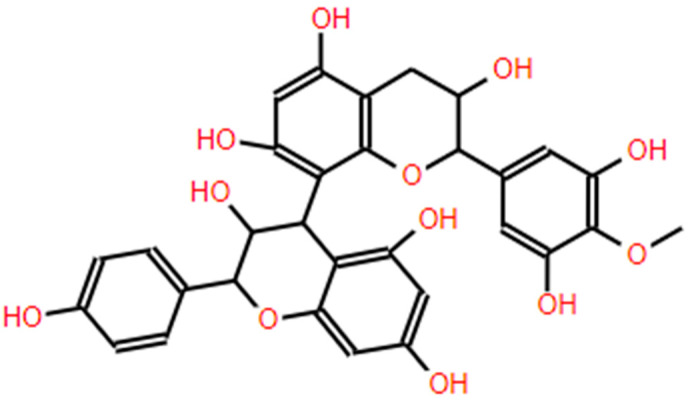
Proanthocyanidin structure.

Proanthocyanidins or leucoanthocyanidins (Figure 3) [48], also known as condensed tannins, are flavonoid polymers present in the seeds and skins of grapes. They consist of flavin units and also contain carbohydrates and traces of amino acids. Proanthocyanidins are polymers of flavan-3-ols linked through C4 to C8 or less frequently C4 to C6 bonds of two consecutive units of varying size and constitution depending on their origin [57,58,59].

Among the properties that can be highlighted with tannins are their high antioxidant capacity, their antimicrobial activity, and their benefits for cardiovascular health by reducing inflammation and improving endothelial function. Proanthocyanidins have been found to improve blood circulation, reduce blood pressure and promote blood vessel health [60,61,62].

However, one compound that has been the subject of numerous studies due to its potential to prevent cardiovascular disease, cancer, and neurodegenerative diseases is resveratrol. This compound was first isolated from *Veratrum grandiflorum*, or white hellebore, in the 1940s [63].

Authors such as Berman et al. (2017) posit that the paucity of clinical evidence casts doubt on the viability of resveratrol as an anticancer therapeutic, further claiming that clinical studies should determine whether the same effects can be observed in human patients.

Resveratrol, with a presence of around 4.58 μg/g in the pomace, has been shown to have positive effects on cognition, cerebrovascular function and bone health in postmenopausal women. Similarly, in patients with polycystic ovary syndrome (PCOS), the anti-inflammatory effect of this compound was evidenced, as well as a decrease in serum levels of (IL)-6, IL-1β, IL-18, TNF-α, NF-KB and YCRP, as well as an improvement in menstrual cyclicity [29,31,33,39]. These studies demonstrated that resveratrol has anti-inflammatory effects through the suppression of NF-κB and NF-κB-regulated gene products.

Other studies confirm that it achieves a decrease in the levels of hemolytic toxicity in patients receiving multi-agent chemotherapy, while in older adults with type 2 diabetes, it decreases markers of oxidative stress and sirtuin. Resveratrol can inhibit pro-inflammatory gene products such as IL-1β, IL-6, and cyclooxygenase 2 (COX-2), and has been shown to reduce blood glucose levels due to the increase in the expression of the glucose transporter (GLUT4) in skeletal muscle cells, improving its uptake, use and storage [30,32].

As can be seen, biowaste from the wine industry is a valuable source of bioactive compounds and contains several organic acids, such as malic acid, tartaric acid and citric acid. These acids contribute to the characteristic sour taste of wines and also have antioxidant properties. Their potential application in functional foods, dietary supplements and pharmaceuticals has aroused great interest in scientific research and the food industry, so extracting these compounds, especially from sources that are potentially discarded, is an issue that is becoming increasingly important.

### 2.3. Current Perspectives on Methods of Extraction and Release of Bioactive Compounds from Grapes

It should be noted that the extraction of polyphenolic compounds can be carried out from fresh, frozen or dried plant samples. In general, before this process, the samples are treated by grinding, crushing and homogenization, which may be preceded by air-drying or freeze-drying, the latter retaining the highest levels of phenolic content [64].

The correct choice of a source is based on the richness of its chemical diversity and its capacity to synthesize compounds with bioactive potential. For this reason, when carrying out an extraction, it is important to take into account the techniques to be used, the solvents, the sources to be studied and the characteristics of the compounds to be extracted. This guarantees a high extraction yield as well as the highest stability of the extracted compounds [46].

In general, extraction is performed using solvents that are capable of separating the desired compounds from a given matrix. The word solvent is derived from the Latin term which generally refers to “to weaken.” These solvents, which are usually fluids with a large structure, are used to decompose, weaken, or suspend substances, and to extract different materials, in most cases without altering either the consistency or the properties of the solvents or the additional materials [65].

In a typical chemical process, solvents are widely used for the dissolution of reactants, to favor the kinetics and thermodynamics of a chemical reaction, for the extraction of products, and for the separation of mixtures. However, most of the organic solvents currently used are characterized by different properties that are detrimental to human health and the environment. It is therefore advocated that solvents should be harmless to man and the environment (safer solvents) and that the substances used in a chemical process should be chosen in such a way as to minimize the potential for chemical accidents (intrinsically safe processes) [66].

Table 1 shows different methods generally used in the extraction of compounds from grapes and their biowaste.

#### 2.3.1. Conventional Extraction Methods: Solid–Liquid Extraction

Solid–liquid extraction is a matter transfer process in which the compound of interest passes from the matrix of the solid into the liquid phase. This process consists of two stages: in the first stage, there is a transfer of the compound from the surface of the solid to the solvent; in the second stage, there is a transfer by diffusion from the interior of the solid to the liquid phase [80].

Solvents such as methanol, ethanol, acetone, ethyl acetate and their combinations, often with different proportions of water, have been used for the extraction of phenols from plant materials. The selection of the appropriate solvent, as well as its polarity, influences the amount and rate of extraction of polyphenols [64].

In the specific case of grape pomace, it is estimated that up to 70% of polyphenols can be found, since there are no effects on the chemical composition during the winemaking process, with the exception of some variation in the composition of carbohydrates during the fermentation of red wine [81]. For this reason, various solvents capable of providing sufficiently high performance have been studied.

Ethanol is a good solvent for the extraction of polyphenols and is safe for human consumption. To prepare anthocyanin-rich phenolic extracts from plant materials, an acidified organic solvent, usually methanol or ethanol, is used. This solvent system denatures cell membranes, simultaneously dissolves the anthocyanins and stabilizes them. However, care must be taken to avoid the addition of excess acid that can hydrolyze the labile, acyl and sugar residues during the concentration steps [64]. The variation in the percentage of ethanol for extraction varies depending on several aspects such as extraction time and temperature. There are reports where ethanol is used at 50%, 60%, 70%, 80% and up to 100% [67,77,82].

The choice of the appropriate solvent also depends on the physical and chemical properties of the solid to be extracted. Some of the solvents commonly used in solid–liquid extraction include the following [83,84,85].

1. Water: It is one of the most widely used solvents due to its wide availability, low toxicity and ability to dissolve a variety of polar compounds. Water extraction is particularly effective for organic and inorganic compounds that are soluble in water.
2. Chloroform: Despite its toxicity, chloroform is used in the extraction of organic compounds due to its high capacity to dissolve a variety of substances. However, its use has been reduced due to environmental and health concerns.
3. Hexane: This organic solvent is ideal for the extraction of non-polar compounds such as fats, oils and aromatics. It is highly volatile and is commonly used in the food and pharmaceutical industries.
4. Acetic acid: It is used in the extraction of acidic or basic organic compounds, since it can form water-soluble salts with these compounds, facilitating their extraction.
5. Methanol: It is known for its high polarity, making it suitable for the extraction of some semi-polar polar compounds. However, it is highly toxic if ingested, inhaled or absorbed through the skin. It is generally used for the extraction of alkaloids, phenols, and other polar compounds.
6. Ethanol: It is highly used for the extraction of polar and semi-polar compounds. It has moderate toxicity, so it is relatively safe in small quantities, but can be toxic in large doses. It is easy to handle and accepted for use in the food and pharmaceutical industry.
7. Acetone: Suitable for a wide range of organic compounds, including polar and non-polar compounds. Its most common use is the extraction of essential oils, resins, and a wide variety of organic compounds. Excellent dissolving capacity for many organic compounds; fast drying due to its low boiling point; but exhibits high flammability and risk of inhalation toxicity.
8. Ethyl acetate: Can be used for a wide range of organic compounds, especially semi-polar and non-polar compounds. With moderate toxicity, it is relatively less toxic than other organic solvents, but can cause irritation and negative effects with prolonged exposure. Its most common uses are the extraction of alkaloids, terpenes, flavonoids, and other semi-polar compounds.

The solvent–solid ratio is a critical aspect in extraction processes, where the aim is to transfer the soluble components of a solid to a liquid solvent. This parameter significantly influences the efficiency and selectivity of the extraction process, as well as the associated costs [86,87].

This ratio is often associated with solid–liquid extraction, but while solid–liquid extraction involves the transfer of soluble components from a solid to a liquid using a solvent, the solvent-to-solid ratio refers to the ratio of solvent to solid in the mixture used in the extraction process. This ratio can vary considerably depending on several factors, including the solubility of the solid in the solvent, the nature of the solid and the solvent, as well as the specific objectives of the extraction.

There are some considerations in the selection of solvent–solid ratio, as follows [88].

- Solubility of the Solid in the Solvent: The solvent-to-solid ratio must be high enough to ensure that the solid is completely dissolved in the solvent. This ensures efficient extraction of the soluble components of the solid.
- Objectives of Extraction: The specific objectives of the extraction process, such as the purity of the final product and the yield of the process, will influence the selection of the solvent-to-solid ratio. In some cases, a higher ratio may be chosen to maximize extraction, while in other cases, a lower ratio may be preferable to minimize costs.
- Practical Considerations: Practical factors, such as the capacity of the extraction equipment, available time and operating costs, also influence the selection of the solvent-to-solid ratio. It is important to find a balance between process efficiency and economic viability.

At the forefront of research, significant innovations have been developed to improve the extraction and release of bioactive compounds. New technologies, such as ultrasound-assisted extraction and nanotechnology, have proven effective in increasing the efficiency of compound release. In addition, specific enzymatic approaches and the use of controlled release systems represent remarkable advances in the optimization of these processes [89,90,91].

#### 2.3.2. Emerging Technologies for the Extraction of Bioactive Compounds—News

##### Enzyme-Assisted Extraction

**Figure 4:**
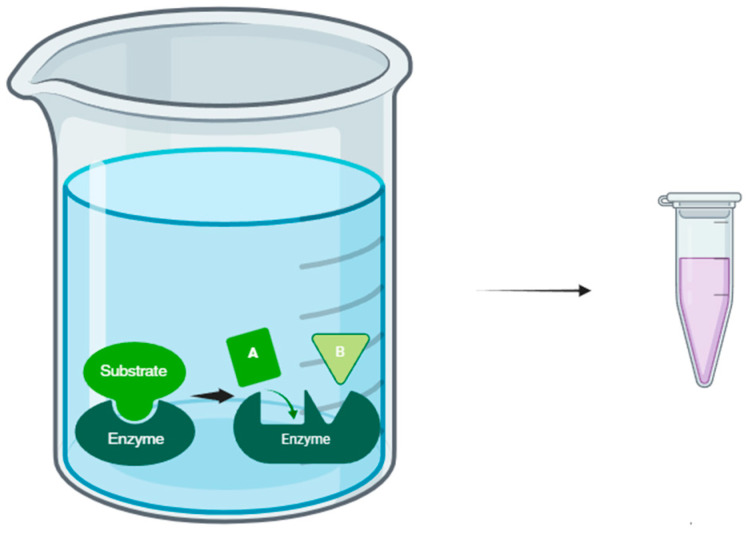
Enzyme-assisted extraction schema.

Enzyme-assisted extraction (EAE) represents an innovative and efficient methodology for the recovery of bioactive compounds and metabolites of interest from raw materials of plant, animal or microbial origin. This approach, based on the catalytic activity of specific enzymes (Figure 4), has gained considerable attention in scientific research and industry due to its ability to improve the selectivity, efficiency and sustainability of extraction processes [92,93].

In recent years, there have been significant advances in the optimization of enzyme-assisted extraction conditions to improve the efficiency and selectivity of the process. These advances include the selection and purification of enzymes with high activity and specificity, the optimization of enzyme reaction conditions, and the development of multifunctional enzyme systems for the simultaneous extraction of multiple compounds [92].

There is evidence that for the recovery of polyphenolic compounds from grape pomace, the enzyme-assisted method exhibits a gradual release of these compounds over time [94]. In addition, the use of emerging technologies, such as ultrasonication, high hydrostatic pressures, microwave irradiation and membrane technology, have been explored to improve the efficiency of enzyme-assisted extraction. These technologies can increase the permeability of the feedstock matrix, accelerate enzymatic reactions and improve mass transfer, resulting in faster and more efficient extraction of the compounds of interest [47,93,94,95].

Enzyme-assisted extraction finds applications in a wide range of industries, including food, pharmaceutical, cosmetic and nutraceutical. Some of the key benefits of this approach include [92]:

- Selectivity: Enzymes can act selectively on specific substrates, allowing the extraction of compounds of interest with high purity and selectivity.
- Efficiency: Enzymatic action can improve extraction efficiency by facilitating the release of compounds from the feedstock matrix in a short time.
- Sustainability: Enzyme-assisted extraction is a green and sustainable process using biodegradable enzymes and mild operating conditions, which reduces energy consumption and waste generated.
- Added value: This approach allows the recovery of bioactive compounds and metabolites with high added value, which can have applications in the formulation of functional products and nutraceuticals.

##### Ultrasound-Assisted Extraction

**Figure 5:**
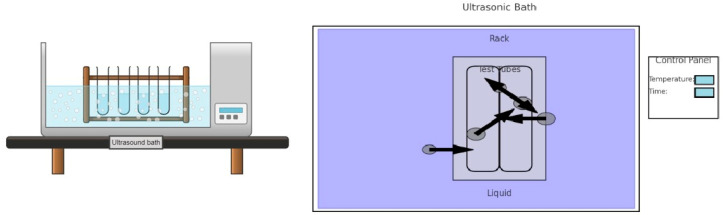
Ultrasound-assisted extraction schema.

Ultrasound-assisted extraction (Figure 5) is based on the principle of acoustic cavitation, where ultrasonic waves generate vacuum bubbles in the extraction medium. These bubbles undergo rapid growth and collapse, creating microcurrents and turbulence that facilitate the rupture of cell structures and the release of soluble compounds into the medium [96].

During the UAE process, the feedstock is mixed with the solvent in a suitable vessel and subjected to ultrasonic irradiation at a specific frequency and controlled power. The ultrasonic energy induces cavitation in the medium, increasing the extraction efficiency and reducing the process time compared to conventional methods.

UAE is applicable in different industries, including food, pharmaceutical, cosmetics, and environmental. Ultrasonic cavitation can increase extraction efficiency by facilitating the breakdown of cellular structures and the release of the compounds of interest in a short time [90].

This approach is more sustainable compared to conventional methods, as it reduces solvent and energy consumption and minimizes waste generated during the process. Being compatible with a wide variety of raw materials and solvents, this extraction method allows the extraction of a wide range of bioactive compounds and metabolites.

##### Supercritical Fluid Extraction

**Figure 6:**
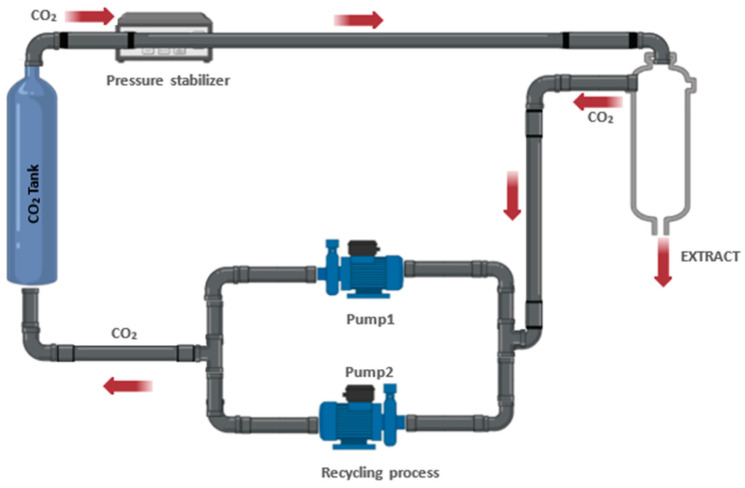
Simplified diagram of supercritical fluid extraction.

Supercritical fluid extraction (SFE) is a state-of-the-art technique that has revolutionized the recovery of essential compounds from various raw materials. This approach uses supercritical fluids, which exhibit properties intermediate between a liquid and a gas, to selectively extract compounds of interest from solid or liquid matrices (Figure 6).

Supercritical fluid extraction is based on the ability of certain fluids, such as carbon dioxide (CO_2_), to exhibit unique properties when above their critical temperature and pressure point. In this state, the supercritical fluid has a high density, low viscosity and fast diffusivity, which makes it highly efficient for the extraction of soluble compounds [96].

During the SFE process, the supercritical fluid is passed through the feedstock matrix, where it comes into contact with the compounds of interest. Pressure and temperature are carefully controlled to optimize the solubility and selectivity of the compounds in the supercritical fluid. Subsequently, the solubilized compounds are recovered by pressure reduction or temperature changes, leaving behind an enriched product.

SFE finds applications in a wide range of industries, including food, pharmaceutical, cosmetics and environmental. Some of the key benefits of this approach include its selectivity since it offers targeted extraction of specific compounds by adjusting the process conditions, such as temperature and pressure of the supercritical fluid. In addition, the most commonly used fluid is CO_2_, which is a non-toxic, non-flammable and readily available solvent, making it a safe and environmentally friendly option for compound extraction [96].

SFE allows the obtaining of high-quality extracts with improved purity and concentration, minimizing the degradation of compounds sensitive to heat or oxidation. This approach is more sustainable compared to conventional methods, as it uses CO_2_ as a solvent, which is recyclable and leaves no residue in the final product [96].

##### Microwave-Assisted Extraction

**Figure 7:**
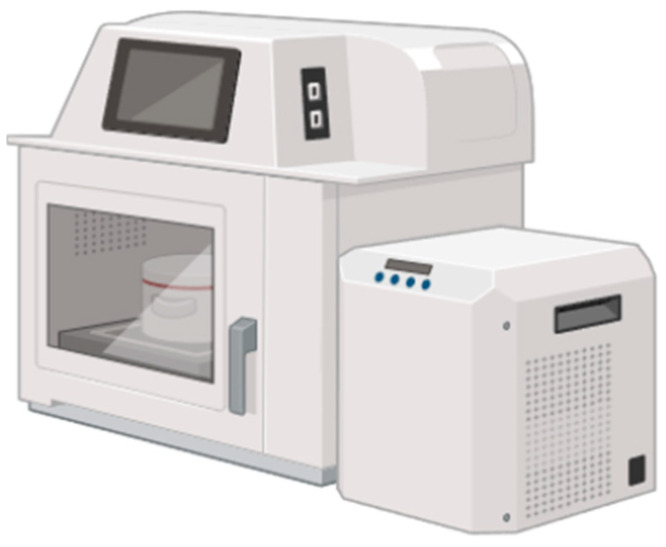
Microwave-assisted extraction schema.

Microwave-assisted extraction (MAE), as shown in Figure 7, has emerged in the field of extraction of bioactive compounds from various raw materials. It uses microwave radiation to selectively heat the solvent and the matrix of the raw material, thus accelerating the extraction process and increasing the recovery efficiency of compounds of interest [75].

Microwave-assisted extraction relies on the ability of microwaves to selectively penetrate the raw material matrix and heat it uniformly through the direct conversion of electromagnetic energy into thermal energy. This rapid and uniform heating increases the solubility of the compounds of interest in the solvent, thus accelerating the extraction process.

During the MAE process, the raw material is mixed with the solvent in a suitable vessel and exposed to microwave radiation at a specific frequency and controlled power. The microwave energy generates heat within the system, facilitating the breakdown of cell structures and the release of soluble compounds into the solvent. The solubilized compounds can then be recovered by conventional separation techniques [96].

MAE allows for faster extraction compared to conventional methods, resulting in increased productivity and reduced process time. However, while it can provide selective extraction of specific compounds by adjusting process parameters such as microwave frequency and power, care must be taken to avoid non-uniform heating and thermal degradation of the compounds [75,96]. This can be seen in studies carried out with grape residues, where it is evident that temperature can influence the final extraction of phenolic compounds [80].

##### Pressurized Liquid Extraction

**Figure 8:**
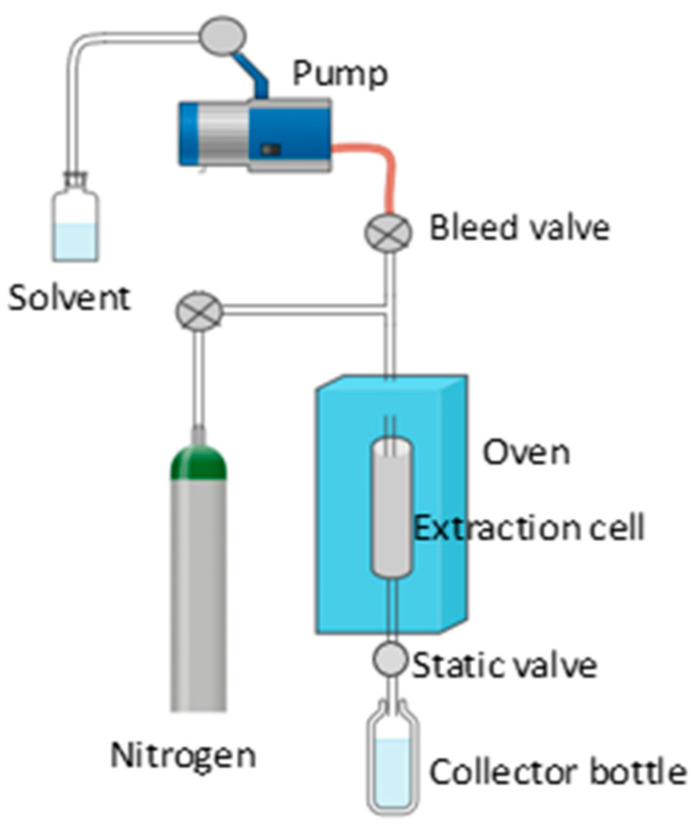
Pressurized liquid extraction schema.

Pressurized liquid extraction (PLE) is an advanced technique that has transformed the recovery of bioactive compounds from various natural sources. This method involves the use of high-pressure liquids as solvents, which increases extraction efficiency and allows the obtaining of high-quality extracts in a reduced time. It is based on the use of liquid solvents at temperatures and pressures above their critical points (Figure 8). Under these conditions, the solvents exhibit unique properties, such as higher density and dissolution capacity, which facilitate the efficient extraction of bioactive compounds from solid or liquid matrices [97,98,99].

During the PLE process, the feedstock is placed in a high-pressure extraction system along with the selected liquid solvent. The pressure is gradually increased until supercritical conditions are reached, where the solvent penetrates the feedstock matrix and solubilizes the compounds of interest. Subsequently, the pressure is reduced to recover the extracts enriched with bioactive compounds [99].

PLE allows rapid and efficient extraction of bioactive compounds compared to conventional methods, resulting in increased productivity and reduced processing time. The solvents used in PLE can be selected to provide selective extraction of specific compounds, thus enabling extracts enriched with compounds of interest to be obtained [97,98].

Since PLE is compatible with a wide variety of raw materials and solvents, it allows for the extraction of a wide range of bioactive compounds and metabolites, and has therefore also been used for the extraction of compounds from grapes and their by-products [98].

##### Hydrostatic High-Pressure Extraction

Extraction by high hydrostatic pressure (HPP) is based on the principle that the application of high pressure increases the solubility of compounds in a solvent, thus allowing for more efficient extraction. During the HPP process, the feedstock is placed in a vessel at high pressure along with the selected solvent. The pressure is gradually increased to specific levels, which facilitates the extraction of bioactive compounds from the feedstock matrix. Subsequently, the pressure is reduced and the enriched extracts are recovered for further analysis or processing [100,101].

HPP enables rapid and efficient extraction of bioactive compounds compared to conventional methods, resulting in increased productivity and reduced processing time [102,103].

**Figure 9:**
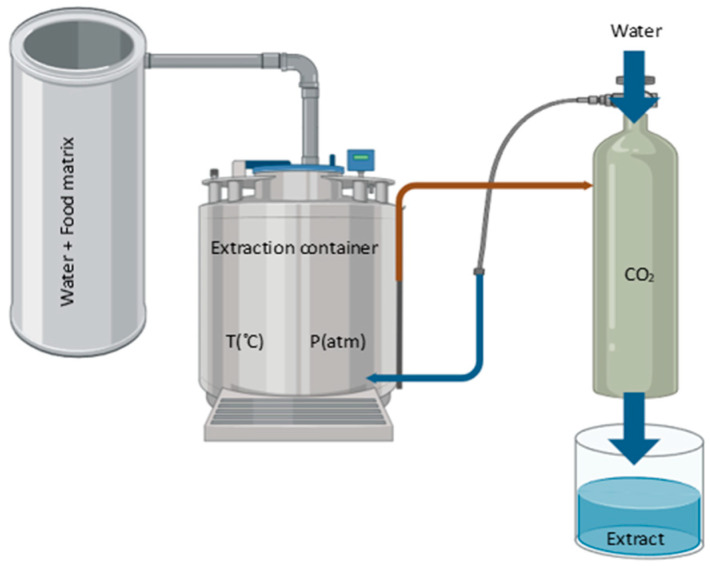
Hydrostatic high-pressure extraction schema.

APH is performed at lower temperatures compared to other extraction methods, which helps preserve the biological activity and nutrients of the extracted compounds [100]. Figure 9 shows a simplified scheme of this extraction method.

##### NaDES-Assisted Extraction

In academic studies and in industry in general, green chemistry is a fundamental objective, with efforts being made to design and synthesize environmentally friendly products as methods to reduce environmental pollution [36].

In search of more environmentally friendly solvents, deep eutectic solvents (DESs) were first used by Abbott et al. in 2003 in a report on the synthesis and study of the viscosities, conductivities, and freezing points of several eutectic mixtures formed with urea and different quaternary ammonium salts [104].

DESs are substances that arise from the mixture between two compounds that, when interacting, result in the formation of a viscous liquid that has a eutectic point, characterized by being lower than the melting point of both compounds individually, and with many similarities to ionic liquids, but more economical and stable in air [105].

Unlike classical ionic liquids composed of components held together by ionic bonding, the components of DESs interact through hydrogen bonds. One of the components acts as a hydrogen bond acceptor (HBA), its partner as a hydrogen bond donor (HBD). The HBA–HBD interaction results in the reduction of the entropy difference of the phase transitions [106,107]. DESs composed strictly of natural components (amino acids, organic acids, sugars or choline derivatives) are often referred to as natural deep eutectic solvents (NaDESs) [108]. Based on their environmental impact, both DESs and NaDESs are considered green solvents. The eutectic character is known in many combinations since the formation of hydrogen bonds is a frequent phenomenon in systems containing hydroxyl and/or carboxyl groups. As for the strength of hydrogen bonds, it correlates completely with the phase transition temperature, stability and dissolution properties of a given eutectic solvent. It is a general rule that the greater the ability to form hydrogen bonds, the greater the decrease in melting temperature. Based on the thermal stability of the compounds selected for the preparation of eutectic solvents, they should be mixed at an elevated temperature that normally does not exceed 100 °C. This temperature limit meets the need for low energy consumption [109].

The use of NaDESs has been studied by researchers, since they are derived from natural products and have excellent properties such as high biodegradability, zero toxicity, sustainability and low cost. Due to their characteristics, the application routes are diverse, covering health, food and cosmetics. In biological systems, NaDESs offer diverse applications: media for enzymatic reactions, biotransformations, extraction procedures, and biomass valorization, among others. In the pharmaceutical and toxicology area, studies have been developed to obtain a higher bioavailability of different low-solubility active compounds [110,111,112,113,114].

##### Discussion

Several studies have focused on the use of these extraction methods, specifically to obtain extracts from grape by-products, from pomace to individual bio-residues such as seeds or skins.

Amendola et al. [67], Garulo-Fuentes et al. [68], and Rodrigues et al. [71] used the solid–liquid extraction method for the extraction of compounds from wine biowaste, highlighting its convenience, especially because it is a simple method, which allows the recovery of different compounds.

Some studies [115] indicate the superiority of water as a solvent compared to ethanol (1.5% and 0.065%, respectively). However, it may depend on the matrix used, as in the case of this research, where the study material was white grape pomace.

Regarding the considerations that exist on solid–liquid extraction, it can be highlighted the fact that for obtaining various compounds, the highest yield is usually found after a prolonged extraction time. A clear example is the work of Bucić-Kojić [41], where after performing a kinetic study, it was shown that the majority of total polyphenols were obtained after 40 min of extraction. This is why the search for options that minimize extraction time, as well as achieve a higher yield, has been redirected to the use of emerging technologies for compound extraction.

In the case of enzymes, since they are capable of separating specific bonds and releasing bioactive compounds, the use of enzymes has aroused the interest of researchers as an assisted form of extraction. However, there are still regulations for their use in the incorporation of processes, due to the formation of components during the process [92].

On the other hand, UAE can provide selective extraction of specific compounds by adjusting the process conditions, such as ultrasonic frequency and solvent type. Its application has been seen in different work, such as that of Lizárraga-Chaidez [42], where after optimizing the process for grape pomace extraction, it was demonstrated that the temperature, amplitude, and time significantly affect the extraction of polyphenolic compounds; however, high-quality extracts are obtained. Another investigation [42] was based on extractions from grape pomace (*Vitis vinifera* L.) of the Tempranillo variety using UAE and MAE. It was determined that at specific conditions of time, temperature and amplitude (15 min, 65 °C, 70%), good extraction efficiency was achieved for polyphenolic compounds. Similarly, there is evidence of the efficacy of UAE in the extraction of oils from grape seeds [116]. The comparison was made against conventional Soxhlet extraction, and the results showed that the extraction time was considerably reduced, not only for oil extraction but also for the extraction of polyphenolic compounds, compared to the maceration method.

UAE has also been used for correlation studies between antioxidant capacity and specific compounds [117] using a 1:1 ethanol–water mixture as solvent.

Using MAE for the extraction of proanthocyanidins from grape pomace, in combination with the use of NaDESs based on choline chloride, satisfactory results have been obtained [118]. Therefore, the control of parameters in this process must be taken into account, as it is certain to be of great advantage. The extraction time was reduced from 1 h to approximately 3.5 min, and the yield was improved from 126 to 135 mgPAC/gGP. The parameters controlled were temperature with MAE of 99.2 °C, 8.3% BM and the combination ChCl–LacA–H_2_O.

Some studies apply ELA in the recovery of value-added compounds from grape residues [119]. Aresta et al. [73] compared the total polyphenol content and extraction yield of different compounds with extracts obtained by a conventional method, with resveratrol showing a positive correlation. Similarly, Porto et al. [74] conducted a study where after a scaling up with satisfactory results, they concluded that with the use of SFE, it is possible to produce high-quality extracts from grape pomace, maintaining the biological activity of the extracts.

NaDESs have high antimicrobial activity compared to conventional solvents such as water and ethanol. And as mentioned above, their low toxicity means that the extracts obtained can be used in food formulation [120].

Punzo et al. [79], studied different combinations of NaDESs based on betaine with citric acid (BET-CA), urea (BET-U) and ethylene glycol (BET-EG). Flavonoid determinations using HPLC-MS/MS showed a similar malvidin content (51–56 μg mL^−1^) in the different combinations.

The use of NaDESs has been tested for the recovery of anthocyanins from grape pomace in combination with hot pressurized water extraction. The results revealed that this was considerably improved, especially when using organic acids such as HBD, specifically choline chloride in combination with oxalic acid and choline chloride with lactic acid [121].

The efficiency of extraction methods for polyphenolic compounds varies according to the technique used due to differences in cell breakdown efficiency, extraction time, costs, and solvent selectivity. Ultrasound- and microwave-assisted extraction improve mass transfer and reduce extraction time, being highly effective in obtaining high yields of polyphenols [122]. High hydrostatic pressures and pressurized liquid allow extraction at low temperatures, preserving the stability of heat-sensitive compounds [123]. Stirring and extraction with NaDEs (natural eutectic solvents) are gentler and more sustainable methods, although they may require more time and optimization to match the efficiency of more intensive techniques. Finally, the use of supercritical fluids, especially CO_2_, allows selective extraction without toxic solvent residues, although with higher cost and technical complexity [124]. These differences in extraction methods reflect the need to choose the appropriate technique according to the specific objectives of yield, quality and sustainability in the extraction of polyphenols.

#### 2.3.3. Preservation of Polyphenolic Extracts—Focus on Microencapsulation

There are different ways to add value to the extracts that can be obtained in the different processes, and one of the most versatile is microencapsulation. This makes it possible to protect polyphenolic compounds from degradation and facilitate their incorporation into foodstuffs.

Microencapsulation is an operation that consists of surrounding a compound (“core”) with an envelope (wall material) that protects it from external factors and releases it under certain conditions. Microencapsulation can be performed using different techniques such as atomization, coacervation, and ionic gelation, among others. One of the most commonly used methods for polyphenolic compounds is spray-drying, which consists of atomizing a solution or suspension containing the extract and the wall material in a stream of hot air, forming dry, spherical particles [125,126,127,128].

There are different substances used for spray-drying. Among them are sodium alginate, which is widely used for encapsulation due to its biodegradability, non-toxicity and its great capacity to control the release depending on the characteristics of the carboxyl group [128]. Sodium casein, on the other hand, has been efficiently used for microencapsulation by spray-drying for the encapsulation of essential oils [129]. Inulin, gum Arabic, or soy protein isolate have also been used in various research studies as wall material in spray-drying [130,131].

One of the most common wall materials is maltodextrin (MD), a starch-derived polysaccharide consisting of multiple D-glucose units linked by α-1,4-glycosidic bonds. The frequent use of this substance is mainly due to its high water solubility, low viscosity and low sugar content. Maltodextrins with a dextrose equivalent between 10 and 20 are widely used to encapsulate anthocyanins and phenolic acids [132,133,134,135,136]. The use of MD has been tested by different authors as an efficient encapsulating material for compounds obtained from grapes or their biowaste, as well as extracts derived from other fruits [137,138,139]. In the case of microencapsulation of polyphenolic compounds extracted with green solvents, the use of chitosan as an encapsulating material has been investigated due to its biocompatible and biodegradable properties. Chitosan is a natural polymer derived from chitin, present in crustaceans, and has been shown to possess loading capacity and controlled release of polyphenolic compound [134]. Microencapsulation, in general terms, is a technique that allows the protection and controlled release of these compounds in various products, improving their stability and bioavailability. The release of bioactive compounds from biowaste is a critical process involving a series of intricate steps. First, enzymatic decomposition and microbial action play a key role in disintegrating the cellular matrices of the waste, thus allowing access to the encapsulated compounds. Subsequently, physical and chemical processes, such as solvent extraction or specific enzymatic techniques, are used to release these compounds from cellular structures and increase their availability. This release process is key to ensure adequate bioaccessibility, directly affecting the biological utility of the compounds in question

The application of bioactive compounds from wine biowaste covers diverse areas, from the food industry to pharmaceuticals. The presence of antioxidants, polyphenols and other beneficial compounds suggests potential applications in the formulation of functional foods, dietary supplements and pharmaceuticals. Ongoing research in this field promises to unlock new possibilities for the application of these compounds for the benefit of human health.

## 3. Conclusions

The bioconversion of wine residues, such as grape pomace and vine shoots, into high-value-added products represents a significant opportunity for sustainability and circular economy in the wine sector. These by-products are rich in polyphenols, flavonoids, anthocyanins and other bioactive compounds, which have potential applications in the food, pharmaceutical and cosmetic industries. Although several studies have explored extraction methods for these compounds, the processes are still being optimized, either by using common solvents, combined methods, or improving already known guidelines.

Among the most prominent techniques for the preservation of polyphenolic extracts, microencapsulation emerges as an effective strategy. This method protects bioactive compounds from degradation and improves their stability and bioavailability, allowing the development of products with longer shelf life and efficacy. Microencapsulation is key in the formulation of supplements and fortified foods, ensuring that the benefits of bioactive compounds remain intact until consumption.

In conclusion, the combination of advanced extraction methods and innovative preservation techniques presents a promising prospect for the valorization of bioactive compounds from wine residues. This synergy not only boosts sustainability and innovation in the industry but also opens up new opportunities for the development of bioactive compounds from wine residues. In addition, the residue generated in these extractions could be used for the formulation of meal for animal consumption, thus completely closing the cycle of this industry.

## Figures and Tables

**Table 1:** Different methods used for the extraction of polyphenolic compounds from grapes and their bio residues.

Extraction Method	Substrate	Advantages	Disadvantages	References
*SLE*	Grape pomace Grape seeds	Simple and widely used method. It requires simple equipment and is suitable for a wide range of samples.	May be less efficient compared to more advanced methods. The duration of the process can be long and some compounds may not be completely extracted.	[67,68]
*EAE*	Grape skins Grape seeds	Selective and gentle. Can improve the extraction of specific compounds without the need for high temperatures. Lower risk of degradation.	Requires specific conditions for each enzyme and can be slower. Enzyme can be expensive and requires precise control of conditions.	[69,70]
*L/S*	Grape pomace	Allows the solvent concentration to be adjusted to suit the sample. Controlling the ratio can influence extraction efficiency.	It may require testing and adjustment to determine the best ratio. In some conditions, an incorrect ratio may adversely affect extraction.	[71]
*UAE*	Grape pomace	Fast and efficient. The use of ultrasound improves mass transfer and can reduce extraction time. Can be used with a variety of solvents.	It can generate heat, which may affect the stability of some compounds. Efficiency may depend on the ultrasonic frequency and power used.	[72]
*SFE*	Grape pomace	It uses supercritical CO_2_, which is safe and evaporates easily. It allows adjustable selectivity and efficient extraction of lipophilic compounds.	It requires specialized equipment and is more expensive. Optimization of conditions is crucial. Not suitable for all compounds.	[73,74]
*MAE*	Grape pomace	Fast and efficient. Microwave energy can accelerate the release of compounds. It can reduce extraction time and improve yields.	Non-uniform energy distribution can result in uneven extractions. Care is needed to avoid thermal degradation of some compounds.	[75]
*PLE*	Grape seeds	Uses pressurized solvents to improve extraction efficiency. Can reduce extraction time and improve yields.	It requires specialized equipment and can be more expensive. Selection of solvent and operating conditions is critical.	[76]
*HHP*	Grape skin	Uses high pressures to improve extraction. Can better preserve the characteristics of heat-sensitive compounds.	It requires specialized equipment and is more expensive. Optimization of pressure conditions is critical. Not suitable for all sample types.	[77]
*NaDES AE*	Grape pomace	NaDES are made from natural components, which gives them a more sustainable profile. They exhibit low toxicity compared to other solvents. They can be designed to be highly selective in the extraction of certain compounds.	NaDES can be expensive to obtain or synthesize compared to other extraction methods. They may require specific temperature, pressure or pH conditions to achieve effective extraction. Research is still ongoing to determine their feasibility on a large scale.	[78,79]

Abbreviations: SLE: solid–liquid extraction; EAE: enzyme-assisted extraction; L/S: solvent-to-solid ratio; UAE: ultrasound-assisted extraction; SFE: supercritical fluid extraction; MAE: microwave-assisted extraction; PLE: pressurized liquid extraction; HHP: high hydrostatic pressure; NaDES AE: NaDES-assisted extraction.
